# Comparative outcomes of open, laparoscopic, and robot-assisted radical cystectomy using IPTW-adjusted trifecta and pentafecta metrics

**DOI:** 10.37349/etat.2026.1002389

**Published:** 2026-07-29

**Authors:** Nobutaka Nishimura, Makito Miyake, Yuki Oda, Takuto Shimizu, Kota Iida, Mitsuru Tomizawa, Kenta Onishi, Shunta Hori, Yosuke Morizawa, Daisuke Gotoh, Yasushi Nakai, Nobumichi Tanaka

**Affiliations:** IRCCS Istituto Romagnolo per lo Studio dei Tumori (IRST) “Dino Amadori”, Italy; ^1^Department of Urology, Nara Medical University, Nara 634-8522, Japan; ^2^Department of Urology, Tane General Hospital, Osaka 550-0025, Japan; ^3^Department of Prostate Brachytherapy, Nara Medical University, Nara 634-8522, Japan

**Keywords:** radical cystectomy, laparoscopic radical cystectomy, robot-assisted radical cystectomy, trifecta, pentafecta

## Abstract

**Aim:**

Minimally invasive approaches for radical cystectomy (RC) have been increasingly adopted; however, comparative evidence regarding surgical quality and perioperative outcomes among open (ORC), laparoscopic (LRC), and robot-assisted RC (RARC) remains limited. This study evaluated these three modalities using standardized composite metrics, trifecta and pentafecta, and inverse probability of treatment weighting.

**Methods:**

We retrospectively analyzed 192 patients who underwent RC with ileal conduit or neobladder reconstruction between 2006 and 2023. Inverse probability of treatment weighting was applied using comprehensive clinicopathological covariates. Trifecta and pentafecta achievements, perioperative parameters, and postoperative complications were compared among the ORC (*n* = 110), LRC (*n* = 38), and RARC (*n* = 44) groups.

**Results:**

RARC achieved the most favorable perioperative and surgical outcomes. Specifically, RARC showed significantly higher rates of several trifecta and pentafecta components than ORC, including fewer positive soft tissue surgical margins (0.0% vs. 5.2%, *P* < 0.01), reduced major complication rates within 90 days (16.1% vs. 44%, *P* < 0.05), and fewer urinary diversion–related sequelae within 12 months (1.5% vs. 13.5%, *P* < 0.05). RARC was also associated with significantly lower blood loss than ORC (380 ± 513 mL vs. 2,913 ± 2,997 mL, *P* < 0.01), as well as lower rates of pelvic abscess, gastrointestinal anastomotic leakage, and obstructive ileus (all *P* < 0.01) than ORC. In contrast, LRC was not superior to ORC in terms of major complications or diversion-related outcomes. Local pelvic recurrence within 12 months did not differ significantly among the three modalities.

**Conclusions:**

RARC may be a favorable surgical option for reducing perioperative morbidity in patients with high-grade bladder cancer, with comparable 12-month local recurrence rates among surgical approaches.

## Introduction

Treatment strategies for high-grade (HG) bladder cancer range widely from endoscopic management, such as transurethral resection of bladder tumors, to radical cystectomy (RC), with or without perioperative systemic therapy. In particular, RC remains the standard curative option for muscle-invasive bladder cancer (MIBC) and selected cases of high-risk non-muscle-invasive disease. Historically, open RC (ORC) has been the conventional approach with long-established oncological outcomes but significant comorbidities and morbidity [1, 2].

Advances in minimally invasive surgical technology have led to the development of novel perioperative management strategies. Laparoscopic RC (LRC) gained widespread adoption in the early 2000s, demonstrating reduced blood loss, shorter hospitalization, decreased wound-related pain, and improved cosmetic outcomes compared with ORC [3, 4]. However, LRC is technically demanding and requires advanced laparoscopic skills and prolonged operative time. Several studies have shown that its learning curve is steep, with optimal outcomes largely restricted to high-volume centers [5, 6].

Robot-assisted RC (RARC) is becoming increasingly prevalent worldwide. Robotic systems provide enhanced dexterity, three-dimensional visualization, and improved ergonomics, facilitating mastery of complex pelvic procedures. High-quality evidence, including the RAZOR randomized controlled trial, has demonstrated that RARC achieves oncological outcomes comparable to ORC, while offering perioperative advantages such as reduced blood loss and lower complication rates [7]. Furthermore, the learning curve for RARC is short and reproducible across institutions [8, 9].

With the expanding adoption of RARC, composite quality metrics such as the “trifecta” and “pentafecta” have been proposed to objectively evaluate surgical success in RC. These metrics consist of key intraoperative and postoperative outcomes, including negative surgical margins, adequate lymph node (LN) yield, absence of HG complications, and early functional recovery [10, 11]. Although multiple studies have assessed pentafecta or trifecta achievement after RARC, comparative evaluations of ORC, LRC, and RARC remain limited. Therefore, in this study, we investigated the pentafecta and trifecta achievement rates and major postoperative complications related to cystectomy across three surgical approaches: ORC, LRC, and RARC.

## Materials and methods

### Patient selection

We conducted a retrospective single-center cohort study at Nara Medical University that included patients diagnosed with HG bladder cancer between January 2006 and October 2023. During the study period, the surgical approach for RC transitioned sequentially from ORC to LRC and then to RARC. For this analysis, patients were categorized according to the surgical approach actually performed within the corresponding institutional adoption period: ORC from January 2006 to December 2014, LRC from January 2015 to May 2019, and RARC from June 2019 onward. Patients who underwent a surgical approach outside its corresponding adoption period were not included in the analysis. Moreover, the analysis included only patients who underwent RC performed by surgeons with at least 10 years of experience in this procedure. Pathological diagnosis was confirmed according to the 2004 World Health Organization classification criteria applied during the study period. Three surgical cohorts were identified: patients who underwent ORC, LRC, and RARC. Baseline patient characteristics including age, sex, smoking history, body mass index (BMI), Eastern Cooperative Oncology Group performance status (ECOG-PS), estimated glomerular filtration rate (eGFR), clinical N stage, administration of neoadjuvant chemotherapy (NAC) or adjuvant chemotherapy (AC), extent of LN dissection, pathological T and N stages, lymphovascular invasion (LVI), and histological subtype were collected. Some patients were excluded if they underwent concomitant nephroureterectomy, pelvic exenteration, ureterocutaneostomy, or did not receive urinary diversion (UD), as the trifecta and pentafecta metrics specifically evaluated the outcomes related to UD. Clinical management, including indications for cystectomy, perioperative care, and surveillance, was conducted in accordance with the institutional standards and contemporary clinical guidelines.

### Outcomes

The surgical quality after RC was evaluated using the established trifecta and pentafecta frameworks [10]. In this study, trifecta was defined by the achievement of three criteria: negative soft tissue surgical margins (STSMs), removal of at least 16 LNs during pelvic lymphadenectomy, and the absence of major postoperative complications, which were classified as Clavien–Dindo grade 3 or higher within 90 days after surgery. To provide a more comprehensive assessment of perioperative quality and early oncological outcomes, we adopted the pentafecta measure, which incorporates all trifecta components, in addition to two further criteria. The five pentafecta items used in this study were as follows:

- Negative STSMs
- Lymphadenectomy with ≥ 16 LNs removed
- Absence of major postoperative complications (Clavien–Dindo ≥ 3) within 90 days
- Absence of UD-related sequelae within 12 months
- No local pelvic recurrence within 12 months after RC

Local recurrence was defined as a radiologically or histologically confirmed tumor relapse within the pelvic cavity.

Postoperative complications were systematically recorded and graded according to the Clavien–Dindo classification, and only complications attributable to cystectomy were analyzed [12, 13]. In addition to overall complication severity, three clinically relevant complications including pelvic abscess, gastrointestinal anastomotic leakage, and obstructive ileus were individually assessed and categorized as major (grade ≥ 3) or minor (grade ≤ 2). The incidences were compared among the three groups. Intraoperative and perioperative parameters were extracted from the operative and anesthetic records, including estimated blood loss, total operative time, pneumoperitoneum duration, and robotic console time.

### Statistical analysis

Inverse probability of treatment weighting (IPTW) based on propensity scores was applied to adjust for baseline imbalances among the three surgical cohorts. Propensity scores were estimated using multinomial logistic regression, incorporating all relevant clinicopathological variables including age, sex, smoking history, BMI, ECOG-PS, eGFR, clinical N stage, administration of NAC and AC, extent of LN dissection, pathological T and N stages, LVI, and histological subtype. Stabilized weights were calculated to minimize the variance inflation. Covariate balance before and after IPTW was assessed using absolute standardized mean differences. Because the exposure variable consisted of three surgical approaches, pairwise SMDs were calculated for each comparison among ORC, LRC, and RARC. The maximum absolute pairwise SMD was used as a summary measure of covariate imbalance for each variable.

After IPTW adjustment, weighted comparisons of trifecta and pentafecta achievement, as well as perioperative complication outcomes, were conducted across the three groups. Overall differences among the three surgical groups were assessed using an omnibus Wald test derived from the IPTW-weighted regression model. Pairwise comparisons were subsequently performed using model-based contrasts from the same weighted regression model in the following order: ORC versus LRC, ORC versus RARC, and LRC versus RARC. For each outcome, pairwise *P* values were adjusted for the three comparisons using Holm’s method. In addition, the presence or absence of major or minor complications and intraoperative parameters were compared using weighted logistic regression models. All statistical analyses were performed using R software (version 4.4.1). Weighted analyses were conducted using packages including WeightIt, survey, cobalt, and tableone. Statistical significance was defined as a two-sided *P*-value of < 0.05. Graphs were generated using GraphPad Prism 10.0 (GraphPad Software, San Diego, CA, USA).

## Results

### Patient selection and IPTW-adjusted characteristics among the three cohorts

Figure S1 shows the patient selection process. Among the 241 patients who underwent RC for HG bladder cancer between January 2006 and October 2023, 17 patients who underwent concomitant nephroureterectomy and nine who underwent pelvic exenteration were excluded. In addition, 17 patients who underwent ureterocutaneostomy and six patients who did not undergo UD were excluded. A total of 192 patients who underwent RC with either ileal conduit or orthotopic neobladder reconstruction were included in this analysis. These patients were categorized into three surgical cohorts: 110 underwent ORC, 38 underwent LRC, and 44 underwent RARC.

Baseline characteristics before and after IPTW are shown in Table 1. Before weighting, several clinicopathological variables differed among the three surgical groups. After IPTW, the balance of several covariates improved; however, residual imbalance remained for some variables, particularly pathological T category. The maximum absolute pairwise SMD for pathological T category remained high after weighting, indicating that differences in pathological stage among the three groups were not fully eliminated by IPTW. Therefore, although IPTW reduced some baseline differences, complete covariate balance was not achieved.

**Table 1:** Clinicopathological characteristics of patients with ORC, LRC, and RARC.

**Variables**	**Unweighted**					**IPTW-adjusted**				
	**ORC**	**LRC**	**RARC**	***P* value**	**Max SMD**	**ORC**	**LRC**	**RARC**	***P* value**	**Max SMD**
**Total**	110	38	44			107.2	34.5	31.6		
**Age, years, mean ± SD**	69 ± 9.2	72 ± 6.8	72 ± 11	0.16	0.37	70 ± 8.8	72 ± 7.5	72 ± 7.5	0.29	0.25
**Sex**				0.27	0.33				0.15	0.58
Male	86 (78%)	29 (76%)	39 (89%)			87.0 (81%)	25.6 (74%)	29.9 (95%)		
Female	24 (22%)	9 (24%)	5 (11%)			20.2 (19%)	8.9 (26%)	1.7 (5.3%)		
**Smoking history**				0.29	0.37				0.36	0.57
Never	27 (25%)	9 (24%)	7 (16%)			22.1 (21%)	8.8 (26%)	14.7 (47%)		
Former/Current	78 (71%)	25 (66%)	36 (82%)			79.2 (74%)	23.0 (67%)	15.9 (50%)		
Unkown	5 (4.5%)	4 (11%)	1 (2.3%)			5.9 (5.5%)	2.7 (7.8%)	1.0 (3.2%)		
**BMI, kg/m^2^, mean ± SD**	22.3 ± 3.5	21.2 ± 3.9	18.2 ± 3.0	< 0.01	1.26	21.4 ± 3.4	21.6 ± 4.4	21.3 ± 3.8	0.99	0.07
**ECOG-PS**				0.05	0.55				0.57	0.41
0	101 (92%)	27 (71%)	40 (91%)			94.0 (88%)	27.7 (81%)	29.7 (94%)		
1	5 (4.5%)	8 (21%)	3 (6.8%)			9.7 (9.0%)	4.5 (13%)	1.6 (5.0%)		
2 ≥	4 (3.6%)	3 (7.9%)	1 (2.3%)			3.6 (3.3%)	2.2 (6.4%)	0.3 (1.0%)		
**eGFR, mL/min/1.73 m^2^, mean ± SD**	65.1 ± 22.9	62.5 ± 25.7	63.1 ± 22.2	0.81	0.11	64.1 ± 23.0	64.2 ± 24.1	68.1 ± 16.5	0.52	0.2
**Clinical N category**				0.53	0.41				0.66	0.34
cN0	98 (89%)	33 (87%)	42 (96%)			97.3 (91%)	31.9 (93%)	31.1 (98%)		
cN1	5 (4.5%)	3 (7.9%)	0 (0.0%)			3.7 (3.5%)	1.0 (2.8%)	0.0 (0.0%)		
cN2	6 (5.5%)	2 (5.3%)	1 (2.3%)			5.5 (5.2%)	1.6 (4.6%)	0.2 (0.8%)		
cN3	1 (0.9%)	0 (0.0%)	1 (2.3%)			0.7 (0.7%)	0.0 (0.0%)	0.3 (0.9%)		
**NAC**				< 0.01	0.63				0.62	0.25
Yes	42 (38%)	22 (58%)	30 (68%)			52.3 (49%)	12.6 (37%)	12.4 (39%)		
No	68 (62%)	16 (42%)	14 (32%)			54.9 (51%)	21.9 (63%)	19.2 (61%)		
**LN dissection**				0.03	0.38				0.57	0.14
Yes	109 (99%)	35 (92%)	40 (91%)			105.6 (99%)	33.2 (96%)	30.4 (96%)		
No	1 (0.9%)	3 (7.9%)	4 (9.1%)			1.6 (1.5%)	1.3 (3.6%)	1.2 (3.8%)		
**Pathological T category**				0.06	0.71				0.49	0.75
pT0	16 (15%)	12 (32%)	15 (34%)			32.3 (30%)	8.3 (24%)	18.6 (59%)		
pTa	1 (0.9%)	1 (2.6%)	0 (0.0%)			0.8 (0.7%)	0.3 (0.8%)	0.0 (0.0%)		
pTis	12 (11%)	3 (7.9%)	4 (9.1%)			9.1 (8.5%)	3.1 (8.9%)	2.9 (9.1%)		
pT1	13 (12%)	3 (7.9%)	3 (6.8%)			9.8 (9.2%)	1.9 (5.5%)	1.7 (5.5%)		
pT2	21 (19%)	6 (16%)	7 (16%)			16.8 (16%)	8.8 (26%)	2.7 (8.5%)		
pT3	23 (21%)	8 (21%)	15 (34%)			20.0 (19%)	7.4 (21%)	5.7 (18%)		
pT4a	23 (21%)	5 (13%)	0 (0.0%)			17.8 (17%)	4.7 (14%)	0.0 (0.0%)		
pT4b	1 (0.9%)	0 (0.0%)	0 (0.0%)			0.6 (0.5%)	0.0 (0.0%)	0.0 (0.0%)		
**Pathological N category**				0.01	0.61				0.4	0.46
pN0	88 (80%)	25 (66%)	39 (89%)			87.5 (82%)	28.2 (82%)	30.0 (95%)		
pN1	11 (10%)	4 (11%)	1 (2.3%)			7.8 (7.3%)	1.6 (4.7%)	0.4 (1.4%)		
pN2	10 (9.1%)	6 (16%)	0 (0.0%)			10.3 (9.6%)	3.4 (9.9%)	0.0 (0.0%)		
Nx†	1 (0.9%)	3 (7.9%)	4 (9.1%)			1.6 (1.5%)	1.3 (3.6%)	1.2 (3.8%)		
**LVI**				0.47	0.21				0.57	0.55
Yes	59 (54%)	18 (47%)	19 (43%)			49.1 (46%)	16.6 (48%)	7.4 (23%)		
No	51 (46%)	20 (53%)	25 (57%)			58.1 (54%)	17.9 (52%)	24.2 (77%)		
**Subtype**				0.43	0.29				0.4	0.24
Yes	12 (11%)	6 (16%)	3 (6.8%)			10.6 (9.8%)	2.4 (7.0%)	1.2 (3.8%)		
No	98 (89%)	32 (84%)	41 (93%)			96.6 (90%)	32.1 (93%)	30.4 (96%)		
**AC**				0.22	0.36				0.63	0.2
Yes	19 (17%)	5 (13%)	12 (27%)			18.6 (17%)	4.5 (13%)	3.3 (11%)		
No	91 (83%)	33 (87%)	32 (73%)			88.4 (83%)	30.0 (87%)	28.3 (89%)		

†: Nx indicates cases in which lymph node dissection was not performed (*n* = 8). AC: adjuvant chemotherapy; ECOG-PS: Eastern Cooperative Oncology Group performance status; eGFR: estimated glomerular filtration rate; LN: lymph node; LRC: laparoscopic radical cystectomy; LVI: lymphovascular invasion; NAC: neoadjuvant chemotherapy; ORC: open radical cystectomy; RARC: robot-assisted radical cystectomy.

### Achievement of trifecta and pentafecta

**Figure 1:**
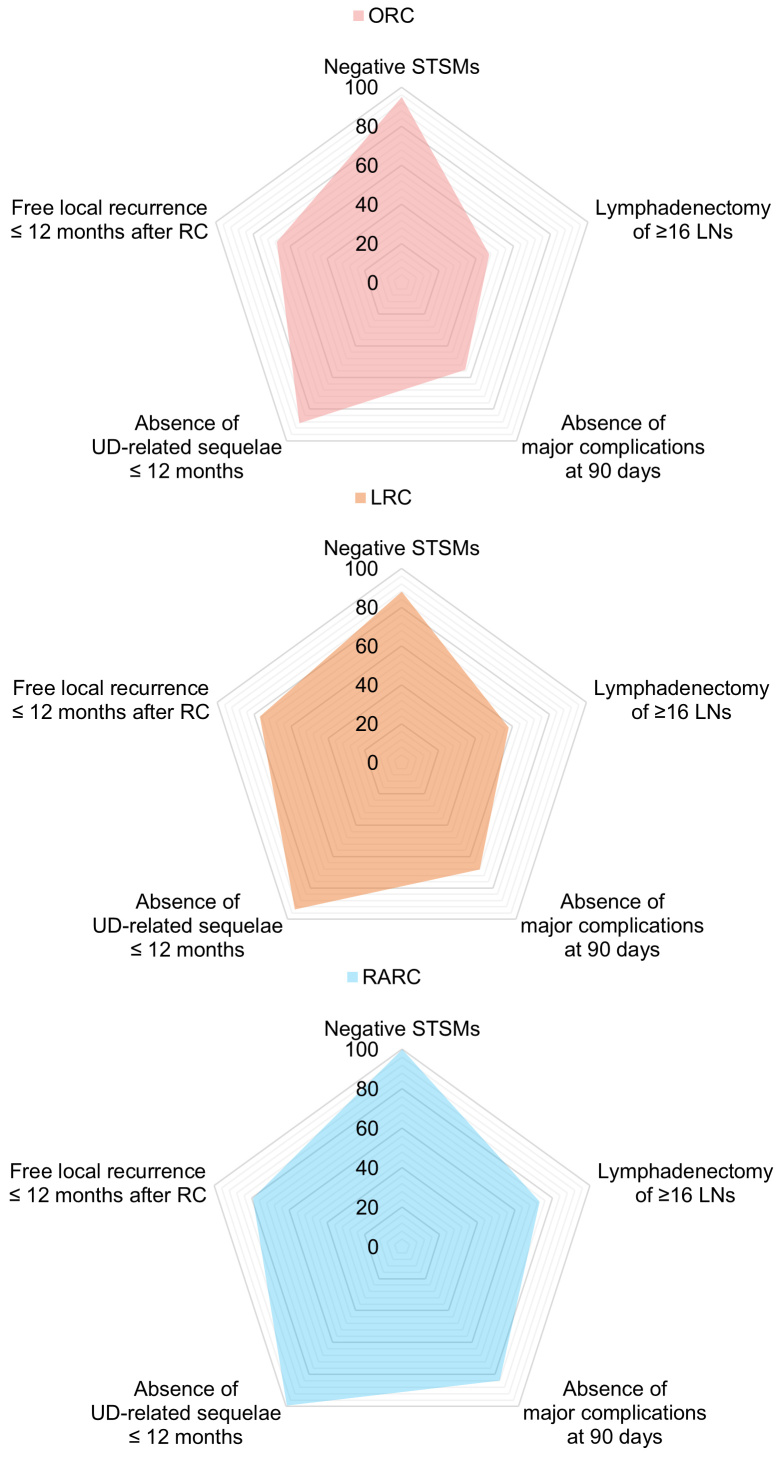
**IPTW-adjusted achievement of pentafecta among the three surgical cohorts.** Distribution of pentafecta achievement after IPTW adjustment among patients who underwent ORC, LRC, or RARC. Pentafecta criteria included: (1) negative STSMs; (2) lymphadenectomy with ≥ 16 lymph nodes removed; (3) absence of major complications within 90 days; (4) absence of UD-related sequelae within 12 months; and (5) no local pelvic recurrence within 12 months after RC. IPTW: inverse probability of treatment weighting; LRC: laparoscopic radical cystectomy; ORC: open radical cystectomy; RARC: robot-assisted radical cystectomy; RC: radical cystectomy; STSMs: soft tissue surgical margins; UD: urinary diversion.

**Figure 2:**
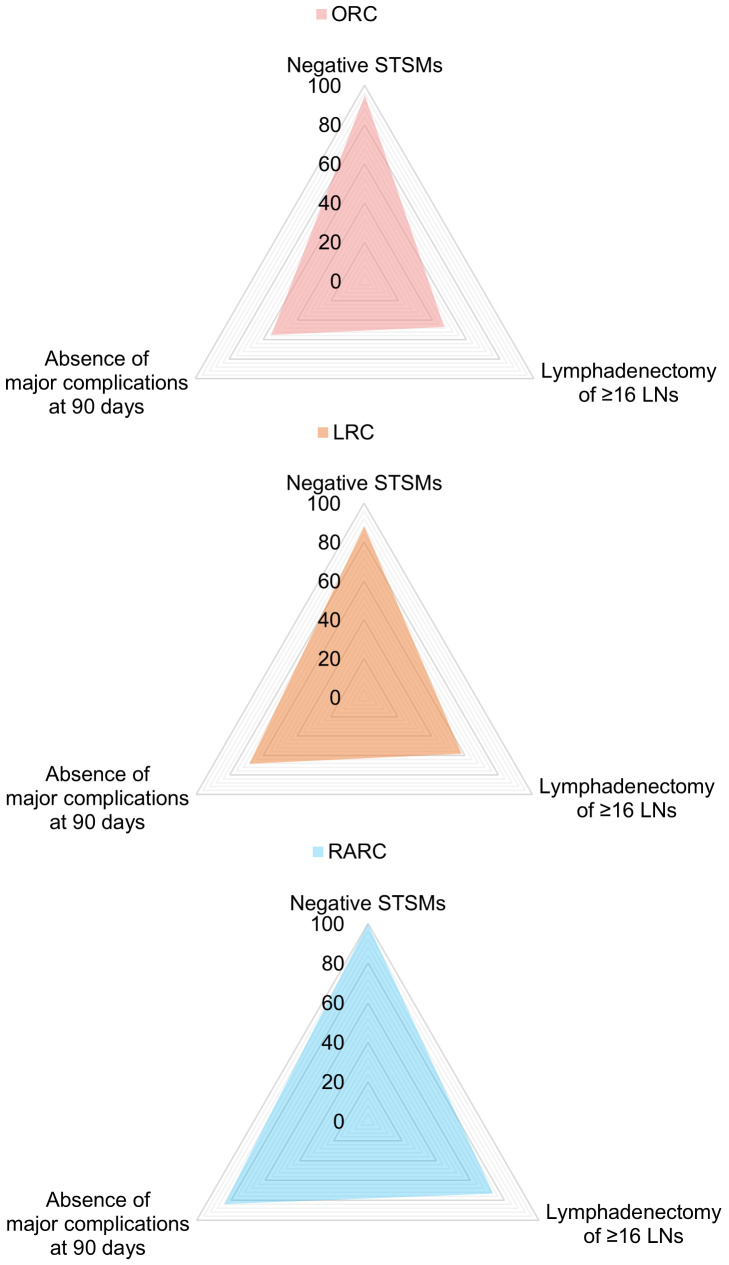
**IPTW-adjusted achievement of trifecta among the three surgical cohorts.** Comparison of trifecta achievement across ORC, LRC, and RARC after IPTW adjustment. Trifecta criteria included: (1) negative STSMs; (2) lymphadenectomy with ≥ 16 lymph nodes removed; and (3) absence of major complications within 90 days after radical cystectomy. IPTW: inverse probability of treatment weighting; LRC: laparoscopic radical cystectomy; ORC: open radical cystectomy; RARC: robot-assisted radical cystectomy; STSMs: soft tissue surgical margins.

**Figure 3:**
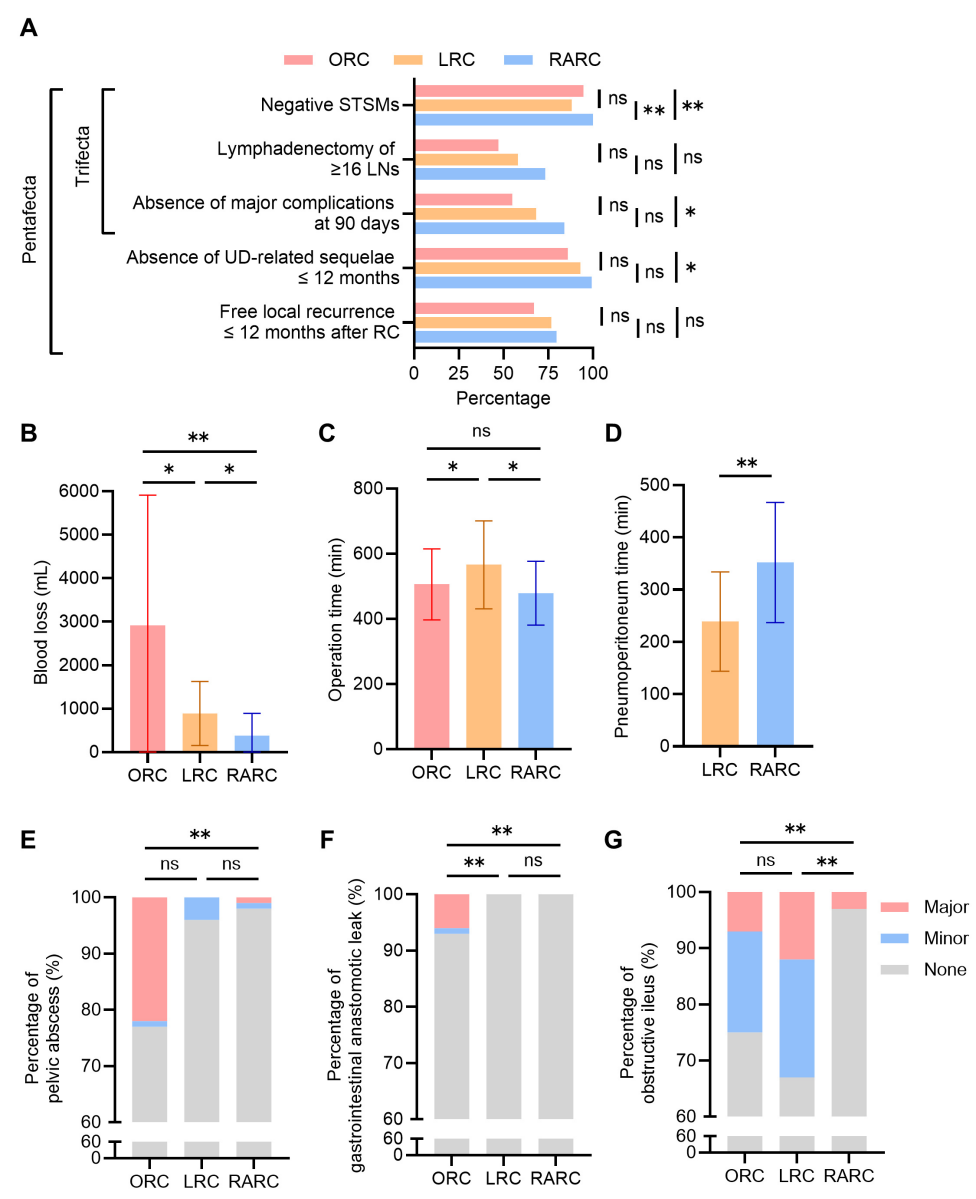
**Detailed comparison of individual trifecta/pentafecta components and peri- and postoperative outcomes.** (**A**) Comparison of each pentafecta and trifecta component after IPTW adjustment among ORC, LRC, and RARC. Components evaluated included STSMs status, removal of ≥ 16 lymph nodes, major postoperative complications within 90 days, absence of UD-related sequelae within 12 months, and absence of local recurrence within 12 months. (**B**–**D**) IPTW-adjusted perioperative outcomes, including estimated blood loss (**B**), operative time (**C**), and pneumoperitoneum duration (**D**) across the three surgical cohorts. (**E**–**G**) IPTW-adjusted postoperative complication rates, including pelvic abscess (**E**), gastrointestinal anastomotic leakage (**F**), and obstructive ileus (**G**). *: Adjusted *P* < 0.05; **: adjusted *P* < 0.01; ns: adjusted *P* ≥ 0.05. IPTW: inverse probability of treatment weighting; LRC: laparoscopic radical cystectomy; ORC: open radical cystectomy; RARC: robot-assisted radical cystectomy; RC: radical cystectomy; STSMs: soft tissue surgical margins; UD: urinary diversion.

Table 2 summarizes the IPTW-adjusted outcomes and Figures 1 and 2 show the trifecta and pentafecta achievements among the IPTW-adjusted cohorts. The pentafecta achievement rates were 27%, 48%, and 64% in the ORC, LRC, and RARC groups, respectively, while the corresponding trifecta achievement rates were 31%, 48%, and 64%, respectively. Figure 3A further describes the distribution of each pentafecta and trifecta component, along with the results of statistical comparisons. Specifically, patients who underwent RARC showed lower rates of positive STSMs (0.0% vs. 5.2%, *P* < 0.01), fewer major complications within 90 days (16.1% vs. 43.6%, *P* < 0.05), and fewer long-term UD-related sequelae within 12 months (1.5% vs. 13.5%, *P* < 0.05). In contrast, LRC did not demonstrate superior outcomes compared with ORC in terms of major postoperative complications or UD-related sequelae. Notably, the incidence of local pelvic recurrence within 12 months after RC did not differ significantly among the three groups.

**Table 2:** IPTW-adjusted outcomes of patients treated with ORC, LRC, and RARC.

**Variables**	**ORC**	**LRC**	**RARC**	***P* value**
**Overall**	107.2	34.5	31.6	
**Operative time category**				
Blood loss, mL, mean ± SD	2,913 ± 2,997	889 ± 738	380 ± 513	< 0.01
Operation time, min, mean ± SD	506 ± 109	566 ± 135	479 ± 98	< 0.01
Pneumoperitoneum time, min, mean ± SD	−	239 ± 95	352 ± 115	< 0.01
Console time, min, mean ± SD	−	−	336 ± 123	NA
**Operative method**				
ICUD				< 0.01
Yes	0 (0.0%)	0 (0.0%)	24.9 (79%)	
No	107.2 (100%)	34.5 (100%)	6.7 (21%)	
UD				0.13
Ileal conduit	102.5 (96%)	34.5 (100%)	27.8 (88%)	
Neobladder	4.7 (4.4%)	0 (0.0%)	3.8 (12%)	
**Outcomes**				
Pentafecta achievement	28.5 (27%)	16.5 (48%)	20.2 (64%)	< 0.01
Trifecta achievement	33.0 (31%)	16.5 (48%)	20.2 (64%)	< 0.01
Positive soft tissue surgical margins	5.6 (5.2%)	4.1 (12%)	0.0 (0.0%)	< 0.05
Number of LNs dissected, mean ± SD	18 ± 10	18 ± 10	18 ± 15	0.99
Lymphadenectomy of ≥ 16 LNs	50.4 (47%)	20.0 (58%)	23.2 (73%)	0.16
Major complications at 90 days	46.7 (44%)	10.9 (32%)	5.1 (16%)	0.09
Long-term UD-related sequelae within 12 months	14.5 (14%)	2.4 (7.1%)	0.5 (1.5%)	0.09
UD-fistulae	0.0 (0.0%)	0.0 (0.0%)	0.0 (0.0%)	NA
UD-urothelial stricture	11.8 (11%)	2.2 (6.3%)	0.2 (0.7%)	0.13
UD-parastomal hernia	3.3 (3.1%)	0.3 (0.8%)	0.3 (0.8%)	0.19
Local recurrence ≤ 12 months after RC	35.3 (33%)	8.0 (23%)	6.5 (21%)	0.45
**Postoperative complications**				
Pelvic abscess				< 0.01
Minor	1.0 (0.9%)	1.2 (3.6%)	0.2 (0.8%)	
Major	23.8 (22%)	0.0 (0.0%)	0.2 (0.7%)	
No	82.5 (77%)	33.2 (96%)	31.2 (99%)	
Gastrointestinal anastomotic leak				NA
Minor	1.2 (1.3%)	0 (0.0%)	0 (0.0%)	
Major	6.5 (6.0%)	0 (0.0%)	0 (0.0%)	
No	99.5 (93%)	34.4 (100%)	31.6 (100%)	
Obstructive ileus				0.18
Minor	18.9 (18%)	7.2 (21%)	0.3 (0.9%)	
Major	7.5 (7.0%)	4.1 (12%)	1.1 (3.4%)	
No	80.8 (75%)	23.1 (67%)	30.3 (96%)	
Paralytic ileus				< 0.01
Minor	19.9 (19%)	2.7 (7.9%)	2.5 (7.8%)	
Major	3.0 (2.8%)	9.5 (28%)	2.2 (7.0%)	
No	84.3 (79%)	22.3 (65%)	26.9 (85%)	

IPTW: inverse probability of treatment weighting; LN: lymph node; LRC: laparoscopic radical cystectomy; ORC: open radical cystectomy; RARC: robot-assisted radical cystectomy; RC: radical cystectomy; UD: urinary diversion.

Additional logistic regression analyses were performed to identify predictors of trifecta and pentafecta achievement (Table 3). In the univariate analyses, NAC administration and RARC were significantly associated with both trifecta and pentafecta achievement (*P* < 0.05, respectively). However, these associations were no longer statistically significant in the multivariable analyses after adjustment for clinically relevant covariates.

**Table 3:** Univariate and multivariate logistic regression analysis for achievement of pentafecta and trifecta.

**Variables**	**Pentafecta**						**Trifecta**					
	**Univariate**			**Multivariate**			**Univariate**			**Multivariate**		
	**ORs**	**95% CI**	***P* value**	**ORs**	**95% CI**	***P* value**	**ORs**	**95% CI**	***P* value**	**ORs**	**95% CI**	***P* value**
**Age at initial TURBT, years**												
< 70	1						1					
≥ 70	1.11	0.60–2.04	0.75				1.08	0.59–1.96	0.81			
**Sex**												
Male	1						1					
Female	0.95	0.45–2.01	0.9				0.86	0.42–1.87	0.75			
**Smoking history**												
Never	1						1					
Former/current	0.7	0.35–1.44	0.33				0.71	0.35–1.43	0.34			
**BMI, kg/m^2^**												
< 22	1						1					
≥ 22	0.72	0.39–1.34	0.3				0.85	0.46–1.54	0.58			
**ECOG-PS**												
0	1						1					
≥ 1	0.73	0.29–1.87	0.52				0.68	0.27–1.75	0.43			
**eGFR, mL/min/1.73 m^2^**												
< 60	1						1					
≥ 60	0.99	0.54–1.83	0.99				1.02	0.56–1.85	0.95			
**NAC**												
No	1			1			1			1		
Yes	1.88	1.02–3.45	0.04	1.68	0.88–3.22	0.11	1.84	1.01–3.34	0.04	1.63	0.87–3.01	0.12
**Surgery**												
ORC	1			1			1			1		
LRC	2.15	0.99–4.64	0.05	1.9	0.82–4.40	0.13	1.89	0.88–4.03	0.1	1.73	0.80–3.74	0.16
RARC	2.21	1.07–4.60	0.03	1.68	0.54–5.18	0.37	1.94	0.95–3.99	0.07	1.69	0.81–3.56	0.17
**UD**												
Ileal conduit	1						1					
Neobladder	1.06	0.29–3.76	0.93				1.26	0.39–4.14	0.69			
**Operation time, min**												
< 480	1						1					
≥ 480	0.97	0.53–1.79	0.92				1.08	0.59–1.97	0.81			
**Blood loss, mL**												
< 500	1			1			1					
≥ 500	0.56	0.29–1.10	0.09	0.82	0.30–2.26	0.71	0.61	0.32–1.19	0.15			
**Pathological T category**												
pT0	1						1					
pTa/pTis/pT1	1.45	0.59–3.52	0.41				1.69	0.71–4.04	0.23			
pT2	0.64	0.24–1.66	0.36				0.64	0.24–1.66	0.36			
pT3	0.67	0.28–1.61	0.37				0.67	0.28–1.61	0.37			
pT4	0.51	0.18–1.46	0.21				0.58	0.21–1.61	0.29			
**Pathological N category**												
pN0	1						1					
pN1	0.59	0.18–1.95	0.39				0.55	0.17–1.80	0.33			
pN2	2.3	0.81–6.54	0.12				2.14	0.76–6.07	0.15			
**LVI**												
No	1						1					
Yes	0.94	0.51–1.70	0.83				0.84	0.46–1.50	0.55			
**Subtype**												
No	1						1					
Yes	0.47	0.13–1.77	0.27				0.44	0.12–1.65	0.23			

ECOG-PS: Eastern Cooperative Oncology Group performance status; eGFR: estimated glomerular filtration rate; LRC: laparoscopic radical cystectomy; LVI: lymphovascular invasion; NAC: neoadjuvant chemotherapy; ORC: open radical cystectomy; RARC: robot-assisted radical cystectomy; TURBT: transurethral resection of bladder tumor; UD: urinary diversion.

### Peri- and post-operative outcomes

Figures 3B–G show detailed comparisons of the peri- and postoperative parameters, including the estimated blood loss, operative time, pneumoperitoneum duration, pelvic abscess, gastrointestinal anastomotic leakage, and obstructive ileus. RARC was associated with significantly reduced blood loss compared with the other two cohorts (mean blood loss: 380 ± 513 mL for RARC vs. 2,913 ± 2,997 mL for ORC and 889 ± 738 mL for LRC). Operative time was longest in the LRC cohort (566 ± 135 min), followed by ORC (506 ± 109 min) and RARC (479 ± 98 min). Pneumoperitoneum time was significantly longer in the RARC group compared with LRC (352 ± 115 min vs. 239 ± 95 min).

Regarding postoperative complications, RARC demonstrated significantly lower rates of pelvic abscess, gastrointestinal anastomotic leakage and obstructive ileus than ORC (major complications: 0.7% vs. 22%, 0.0% vs. 6.0% and 3.4% vs. 7.0%, respectively). In contrast, the frequency of obstructive ileus was highest in the LRC group.

## Discussion

In the present study, RARC was associated with favorable perioperative performance compared with ORC and LRC, as reflected by higher rates of trifecta and pentafecta achievement. RARC was also associated with reduced blood loss, shorter operative time than LRC, and a lower incidence of major postoperative complications. These findings suggest that RARC may offer potential perioperative advantages in selected patients undergoing RC. Previous large randomized trials have reported comparable oncological outcomes between RARC and ORC, and our results similarly showed no significant differences in 12-month local recurrence among the three surgical modalities [14]. However, because the follow-up duration of the RARC cohort was relatively short, these oncological findings should be interpreted as short-term observations rather than definitive evidence of long-term oncological equivalence.

Although the RARC group showed higher trifecta and pentafecta achievement rates, surgical approach was not an independent predictor of these outcomes after multivariable adjustment. This suggests that the observed differences may not be attributable to the surgical approach alone, but may partly reflect differences in patient background, NAC administration, pathological stage, institutional experience, and chronological era effects. Therefore, while the present findings support the potential value of RARC as a favorable surgical option, they should be interpreted cautiously and should not be regarded as definitive evidence of the independent superiority of RARC itself.

A notable finding in the LRC cohort was the lower rate of negative STSMs compared with the other groups. Laparoscopic cystectomy is technically demanding, and the learning curve may substantially influence oncological precision [5, 6]. Although previous reports suggest that positive-margin rates in LRC can be comparable to those in ORC in experienced centers, the markedly higher success rate achieved with RARC in our cohort underscores the technical advantages of robotic articulation, enhanced visualization, and instrument stability [15, 16]. These factors collectively support the preferential adoption of RARC over conventional laparoscopy, especially in institutions where the case volume or surgeon’s experience with pure laparoscopy is limited.

The prolonged pneumoperitoneum time observed in RARC is most likely attributable to our center’s preference for intracorporeal UD (ICUD), which requires additional robotically performed reconstructive steps. Although a longer pneumoperitoneum could theoretically affect postoperative recovery or bowel function, our findings showed that RARC exhibited the lowest rates of gastrointestinal anastomotic leakage and pelvic abscesses, suggesting that the quality of minimally invasive reconstruction offsets any potential disadvantages associated with a longer insufflation duration [17, 18].

Another important observation in this study was the substantially lower frequency of UD–related sequelae within 12 months in the RARC cohort. Minimally invasive manipulation, particularly in ICUD, may reduce bowel manipulation, minimize traction injury, and preserve mesenteric integrity, thereby decreasing the risk of long-term obstruction, ureteroenteric stricture, or other diversion-related complications [19–21]. These early functional advantages are clinically meaningful, as UD morbidity is a major determinant of the postoperative quality of life. The consistent superiority of RARC in both perioperative and diversion-related outcomes suggests that robotic surgery not only facilitates safer intra-abdominal handling, but may also contribute to more durable functional recovery after RC.

In addition to surgical approach, patient- and procedure-related factors may also influence perioperative and oncological outcomes after RC. Recent evidence has suggested that cumulative smoking exposure and smoking cessation status are significantly associated with postoperative complications after RARC [22]. In the present study, smoking history was included as a baseline covariate; however, detailed information on pack-years and time since smoking cessation was not available. Therefore, the impact of cumulative smoking exposure on perioperative morbidity could not be fully evaluated. Furthermore, intraoperative oncological quality-control measures may also contribute to surgical quality after RC. Recent evidence suggests that intraoperative frozen section analysis of distal ureteric margins may help achieve cancer-free anastomosis and guide postoperative surveillance, particularly in selected high-risk patients [23]. Although these factors were not directly incorporated into the present trifecta/pentafecta definitions, future studies including such patient- and procedure-related variables may allow a more comprehensive assessment of surgical quality after RC.

This study had several limitations. First, its retrospective, single-center design introduced inherent biases that could not be fully eliminated despite rigorous statistical adjustments. Although IPTW was performed to adjust for baseline differences, covariate balance was not fully achieved. In particular, residual imbalance remained for pathological T category, which may have influenced surgical margin and recurrence-related outcomes. Therefore, the observed favorable outcomes in the RARC group should be interpreted cautiously, as they may partly reflect differences in disease stage rather than the effect of surgical modality itself. Second, the three surgical approaches were performed during different calendar periods, with ORC mainly performed in the earlier period and RARC in the later period. Therefore, improved outcomes in the RARC group may partly reflect advances in perioperative care, institutional protocols, surgical team experience, and patient management over time, rather than the effect of the robotic approach itself. Although IPTW analysis was performed to adjust for measured baseline differences, statistical adjustment cannot fully eliminate this period effect because surgical modality and operative era were closely correlated. Accordingly, the observed association between RARC and improved trifecta/pentafecta achievement should be interpreted with caution. Third, the surgeries were performed by multiple surgeons over a long study period, and variability in surgeons’ experience, technical proficiency, and learning curves may have influenced the perioperative and oncological outcomes. Fourth, the sample size, particularly for the LRC and RARC cohorts, was relatively small, which may have limited the statistical power to detect differences in less frequent outcomes such as rare complications or early recurrence. Therefore, the corresponding *P*-values may be unstable, and these analyses should be regarded as exploratory rather than confirmatory. Another limitation is the relatively small sample size of the LRC and RARC cohorts, which limited the statistical power, particularly for less frequent outcomes. Although pairwise comparisons showed significantly lower rates of 90-day major complications and urinary diversion-related sequelae in the RARC group than in the ORC group, the corresponding overall comparisons among the three surgical groups did not reach statistical significance. These findings may have been underpowered, and the corresponding *P* values should therefore be interpreted cautiously. Larger multicenter studies are needed to validate these results. Finally, the follow-up duration for assessing the oncological endpoints was limited to 12 months, and long-term comparative analyses are required to determine whether the perioperative advantages of RARC translate into improved survival or durable functional benefits.

## Abbreviations

AC: adjuvant chemotherapy
BMI: body mass index
ECOG-PS: Eastern Cooperative Oncology Group performance status
eGFR: estimated glomerular filtration rate
HG: high-grade
ICUD: intracorporeal urinary diversion
IPTW: inverse probability of treatment weighting
LN: lymph node
LRC: laparoscopic radical cystectomy
LVI: lymphovascular invasion
NAC: neoadjuvant chemotherapy
ORC: open radical cystectomy
RARC: robot-assisted radical cystectomy
RC: radical cystectomy
STSMs: soft tissue surgical margins
UD: urinary diversion

## Supplementary materials

The supplementary figure for this article is available at: https://www.explorationpub.com/uploads/Article/file/1002389_sup_1.pdf.

## Declarations

### Availability of data and materials

The raw data supporting the conclusions of this manuscript will be made available by the authors without undue reservation to any qualified researcher.

### Copyright

© The Author(s) 2026.
