# Supplementary figures and images for: Comparative outcomes of open, laparoscopic, and robot-assisted radical cystectomy using IPTW-adjusted trifecta and pentafecta metrics
Source: Explor Target Antitumor Ther. 2026 Jul 29;7:1002389. doi: 10.37349/etat.2026.1002389 (PMC13424797; doi:10.37349/etat.2026.1002389)

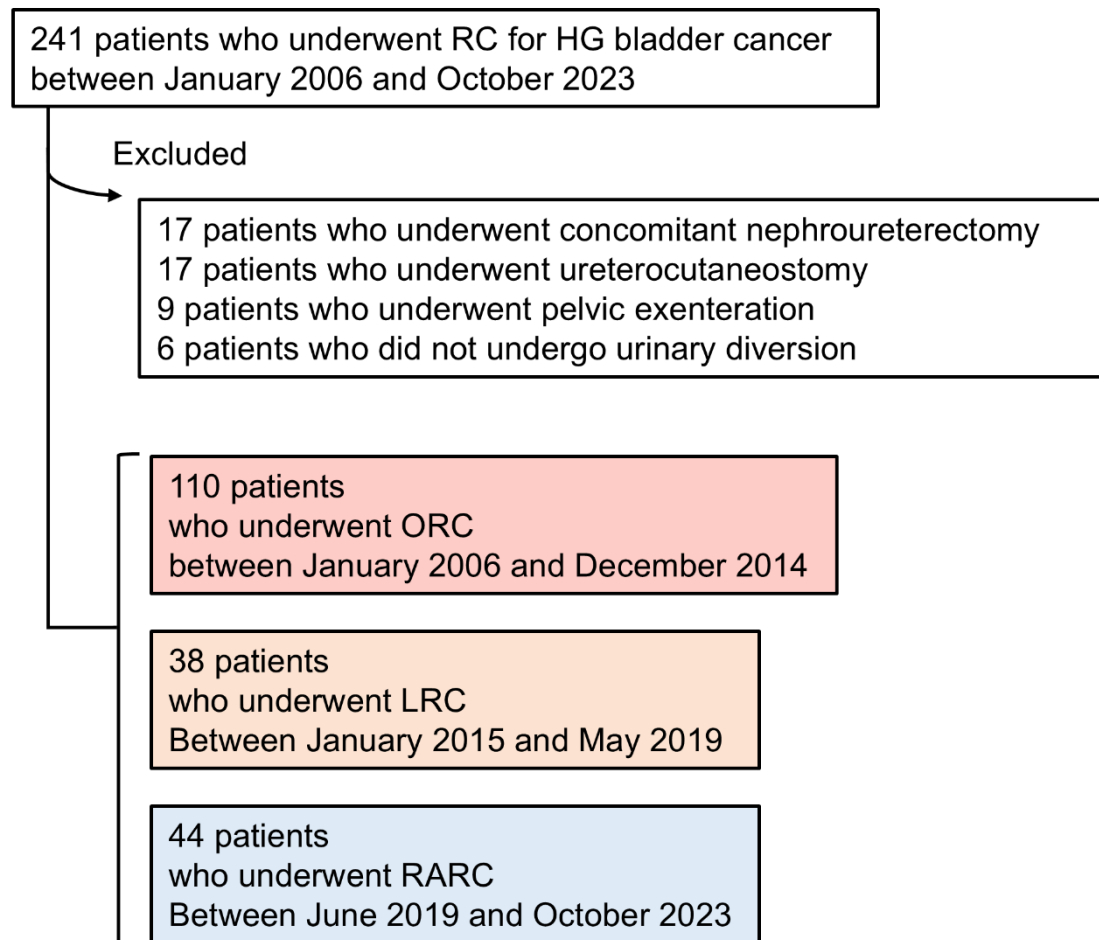

**Figure S1**

Supplement: Supplementary file 1 [file 1002389_sup_1.pdf]
